# High and intermediate grade ductal carcinoma in-situ of the breast: a comparison of pathologic features in core biopsies and excisions and an evaluation of core biopsy features that may predict a close or positive margin in the excision

**DOI:** 10.1186/1746-1596-4-26

**Published:** 2009-08-19

**Authors:** Oluwole Fadare, Nathan F Clement, Mohiedean Ghofrani

**Affiliations:** 1 Department of Pathology, Wilford Hall Medical Center, Lackland Air Force Base, San Antonio, Texas, USA; 2 Department of Pathology, University of Texas Health Science Center at San Antonio, San Antonio, Texas, USA; 3 Department of Pathology & Laboratory Services, Brooke Army Medical Center, Ft Sam Houston, San Antonio, Texas, USA; 4 Pathology Program, San Antonio Uniformed Services Health Education Consortium, San Antonio, Texas, USA; 5 Department of Pathology, Southwest Washington Medical Center, Vancouver, WA, USA

## Abstract

Low and high-grade ductal carcinoma in-situ (DCIS) are known to be highly disparate by a multitude of parameters, including progression potential, immunophenotype, gene expression profile and DNA ploidy. In this study, we analyzed a group of intermediate and high-grade DCIS cases to determine how well the core biopsy predicts the maximal pathology in the associated excisions, and to determine if there are any core biopsy morphologic features that may predict a close (≤ 0.2 cm) or positive margin in the subsequent excision. Forty-nine consecutive paired specimens [core biopsies with a maximal diagnosis of DCIS, and their corresponding excisions, which included 20 and 29 specimens from mastectomies and breast conserving surgeries respectively] were evaluated in detail. In 5 (10%) of 49 cases, no residual carcinoma was found in the excision. In another 4 cases, the changes were diagnostic only of atypical ductal hyperplasia. There were 4 and 3 respective cases of invasive and microinvasive carcinoma out of the 49 excision specimens, for an overall invasion frequency of 14%. In 28 cases where a sentinel lymph node evaluation was performed, only 1 was found to be positive. Among the 40 cases with at least residual DCIS in the excision, there were 5 cases in which comedo-pattern DCIS was present in the excision but not in the core biopsy, attributed to the lower maximal nuclear grade in the biopsy proliferation in 4 cases and the absence of central necrosis in the 5^th^. For the other main histologic patterns, in 8 (20%) of 40 cases, there were more patterns identified in the core biopsy than in the corresponding excision. For the other 32 cases, 100%, 66%, 50%, 33% and 25% of the number of histologic patterns in the excisions were captured in 35%, 5%, 17.5%, 15% and 7.5% of the preceding core biopsies respectively. Therefore, the core biopsy reflected at least half of the non-comedo histologic patterns in 77.5% of cases. In 6(15%) of the 40 cases, the maximum nuclear grade of the excision (grade 3) was higher than that seen in the core biopsy (grade 2). Overall, however, the maximum nuclear grade in the excision was significantly predicted by maximum nuclear grade in the core biopsy (p = 0.028), with a Phi of 0.347, indicating a moderately strong association. At a size threshold of 2.7 cm, there was no significant association between lesional size and core biopsy features. Furthermore, the clear margin width of the cases with lesional size ≤ 2.7 cm (mean 0.69 cm) was not significantly different (p = 0.4) from the cases with lesional size > 2.7 cm (mean 0.56 cm). Finally, among a variety of core biopsy features that were evaluated, including maximum nuclear grade, necrosis, cancerization of lobules, number of tissue cores with DCIS, number of DCIS ducts per tissue core, total DCIS ducts, or comedo-pattern, only necrosis was significantly associated with a positive or close (≤ 0.2 cm) margin on multivariate analysis (Phi of 0.350). It is concluded that a significant change [to invasive disease (14%) or to no residual disease (10%)] is seen in approximately 24% of excisions that follow a core biopsy diagnosis of intermediate or high-grade DCIS. Core biopsy features are of limited value in predicting a close or positive margin in these lesions.

## Introduction

Ductal carcinoma in-situ (DCIS) of the breast is comprised of a heterogeneous spectrum of intraductal epithelial proliferations that are considered the probable precursor lesions to most invasive breast carcinomas [1,2]. The widespread implementation of screening mammography programs has resulted in a dramatic increase in the incidence of DCIS during the past few decades [3-8]. Whereas most DCIS in the pre-mammographic eras were of the comedo-type, screening-detected DCIS tend to be of a comparatively smaller size and lower grade [9]. Although low-grade and high-grade are unified by the fact that both are intraepithelial proliferations that are breast cancer precursors, they are considered to be substantially different processes. Low-grade DCIS is generally positive for the estrogen & progesterone receptors (ER & PR) and negative for HER2/neu, displays chromosomal losses at 16q, gains in 1q and near euploidy [10,11]. High-grade DCIS, in contrast, tends to display lack of expression of ER and PR, HER2/neu overexpression/amplification, a multitude of chromosomal changes, and aneuploidy [10,11]. Expectedly, intermediate grade DCIS displays changes that are intermediate between these two extremes [10]. Detailed evaluations of the protein expression patterns of DCIS of various grades and a comparison of such patterns with those of their synchronous invasive cancers, typically show strong correlations in a grade-dependent pattern [12]. Furthermore, progression from low-grade to high-grade DCIS is considered to be, at most, a very infrequent event [13]. Therefore, in one contemporary model of the evolution of invasive ductal breast carcinomas, well-differentiated ductal carcinomas evolve from low-grade DCIS whereas poorly differentiated invasive ductal carcinomas evolve from high-grade DCIS, with minimal, if any, overlap [11,13]. The present study focused on DCIS lesions that are not on the lower end of the spectrum to obtain a better understanding of this specific group.

Several aspects of the management of patients with DCIS have engendered significant discussions in the recent medical literature, most notably (for the present purposes), the excision modality (breast conserving surgery versus mastectomy), the necessity of adjuvant radiotherapy and the necessity of sentinel lymph node sampling. [14-17]. Some of these controversies are centered, at least in part, on concerns about the ability of pre-definitive surgery measures, such as imaging and core biopsy, to predict the maximal pathology in the patient (i.e. maximal DCIS grade, presence or absence of stromal invasion, and hence axillary lymph node status). In this study, we analyze a group of intermediate and high-grade DCIS cases to determine how well the core biopsy predicts the maximal pathology in the associated excisions, and to determine if there are any core biopsy morphologic features that may predict a close or positive margin in the subsequent excisions.

## Methods

### Core Biopsies

**Figure 1:**
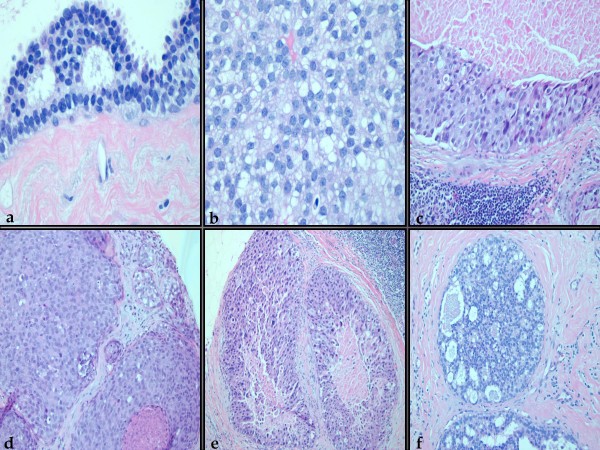
**a-c: Three-tiered nuclear grading system showing grade 1(1a), grade 2 (1b) and grade 3 (1c) cytologic features**. d: Solid pattern ductal carcinoma in-situ with grade 2/3 atypia and microcalcification (upper left field), and solid pattern ductal carcinoma in-situ with central necrosis and nuclear grade 2/3 (lower right field). e: Solid pattern ductal carcinoma in-situ with grade 3/3 atypia and central necrosis (comedo-pattern ductal carcinoma in-situ). f: Cribriform pattern ductal carcinoma in-situ with grade 2/3 atypia and foci of necrosis.

**Figure 2:**
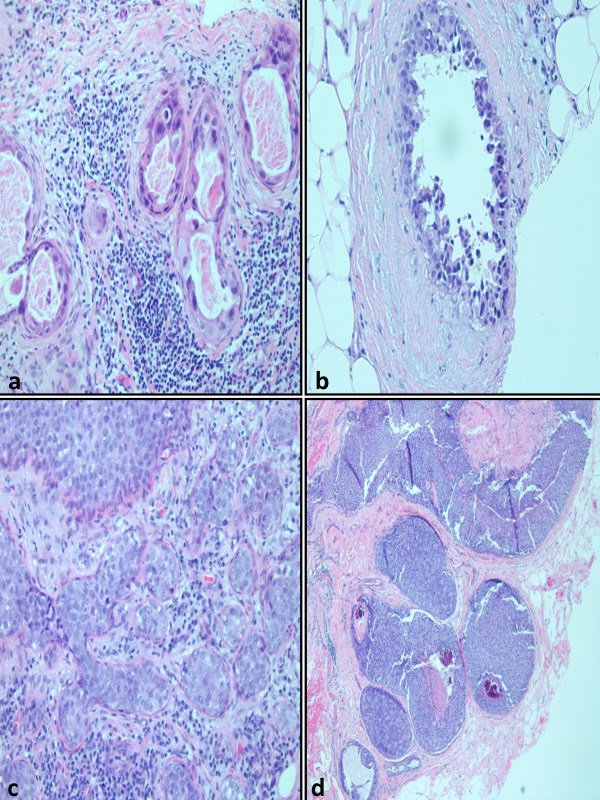
**a: Clinging pattern ductal carcinoma in-situ**. b: Micropapillary pattern ductal carcinoma in-situ. c: Cancerization of lobules by ductal carcinoma in-situ. d: As an example of the counting system for ductal carcinoma in-situ ducts, 6 ducts would be counted in this microscopic field.

Following approval from our institutional review board (protocol FWH 20090088H), the computerized pathologic database of Wilford Hall Medical Center (Lackland AFB, TX) was searched for all core breast biopsies diagnostically coded as ductal carcinoma in situ for the period between January 2006 and January 2009. Cases were excluded if a concurrent invasive or microinvasive carcinoma was diagnosed in the core biopsy and/or a follow-up excision was not available in our records for review. All slides for the core biopsies were reviewed in detail by 2 authors (OF and NFC), and the following items were recorded for each case: 1) range of nuclear grades and maximal nuclear grade (figure 1a-c); 2) histologic patterns of DCIS (i.e. solid, micropapillary, cribriform, clinging, papillary etc, figures 1d-f &2a-b); 3) Presence or absence of central necrosis; 4) Presence or absence of DCIS-associated calcification; 5) Number of tissue cores obtained, as counted on the glass slides; 6) Number of cores with at least one focus with changes diagnostic of DCIS, 7) Presence or absence of lobular cancerization by DCIS (figure 2c); 8) Number of DCIS ducts (ductal cross-sectional profiles of DCIS, *vida infra*; figure 2d); 9) Type of radiological guidance for biopsy. Nuclear grade was assigned using a modification of the 3-tiered scale employed in the modified Scarff Bloom Richardson (MSBR) system for grading invasive breast cancers [18]. Grade 1 nuclei were generally monomorphic and displayed rounded contours, evenly dispersed chromatin whose overall effect is to result in a vaguely hyperchromatic appearance, and inconspicuous nucleoli (figure 1a). Grade 3 nuclei displayed marked nuclear pleomorphism, and cases with grade 3 nuclei were typically characterized by a 3-fold or greater variation in nuclear size and shape (figure 1c). Grade 2 nuclei displayed prominent nucleoli, irregular distributed chromatin and a level of pleomorphism that was intermediate between grade 3 and grade 1 nuclei (figure 1b). Cross sectional profiles of DCIS were counted individually in every tissue core and added (figure 2d). When it could be clearly determined that a focus of interest represented DCIS cancerization of a single terminal ductolobular unit (figure 2c), only 1 cross sectional profile was counted for the whole focus. Unless otherwise specified, the term "solid pattern" is used herein as a generic descriptor for an intraductal epithelial proliferation that fills the duct and which lacks micropapillary and cribriform areas, irrespective of its other features. As such, it encompasses the lesions that have traditionally being referred to as displaying the "comedo-pattern DCIS" or "comedo-DCIS". Comedo-pattern DCIS was defined as solid pattern DCIS with central necrosis and grade 3/3 atypia (figure 1e). By definition, this study only included cases with intermediate or high-grade DCIS in the core biopsy and/or the corresponding excision. Cases of intermediate grade DCIS were characterized by grade 1/3 nuclear atypia with necrosis, or grade 2/3 nuclear atypia irrespective of necrosis status. Cases of high-grade DCIS were characterized by grade 3/3 nuclear atypia with or without necrosis.

### Excisions

Slides for the excisions associated with the aforementioned core biopsies were similarly reviewed. The following items were recorded for each case: 1) range of nuclear grades and maximal nuclear grade; 2) histologic patterns of DCIS, 3) Presence or absence of central necrosis in an otherwise solid pattern; 4) Presence or absence of necrosis in other histologic patterns; 5) Presence or absence of DCIS-associated calcification; 5) Type of excision (breast-conserving surgery [BCS] or mastectomy); 7) Specimen size in 3 dimensions; 8) Number of paraffin-embedded blocks of breast tissue processed; 9) Number of processed blocks with DCIS; 10) Size of DCIS, which was determined using the recently reported findings of Dadmanesh et al [19] - 0.4 cm multiplied by number of blocks with DCIS; 11) Presence or absence of invasive or microinvasive carcinoma; 12) regional lymph node status; 13) Duration between biopsy and excision; 14) Margin status, with a "clear" margin width defined as the linear distance, as measured microscopically, between a DCIS duct and the closest inked margin for that case. All microscopic measurements were made on a BX45 microscope (model U-DO3; Olympus Corporation, Tokyo, Japan). In cases with "positive" margins, the aforementioned "linear distance" was 0 cm. A "close" margin was defined as ≤ 0.2 cm.

### Statistics

Statistical analyses were performed using SPSS for Windows, release 11.5.0 (SPSS Inc., Chicago, IL). Descriptive statistics were prepared for the above mentioned variables. Strength of association between individual variables recorded in the core biopsy and excision (BCS and mastectomy) were measured using Pearson chi-square, Fisher's exact test, and Phi. In order to determine whether any single variable or group of variables predicts a close margin in subsequent excision, linear regression and binary logistic regression were used. Associational analyses were performed on all qualifying cases with residual carcinoma in the subsequent excision, as well as on cases separated into BCS and mastectomy groups.

## Results

A total of 49 paired biopsy/excisions specimens ("cases") from 47 patients were reviewed (2 patients each with bilateral disease and hence bilateral specimen pairs). There was an average of 33.4 (± 11) days between biopsies and excisions (10-72; median 30).

### Core Biopsies

Core biopsies were ultrasound and stereotactic-guided in 7 and 41 cases respectively. Guidance was unknown in the 49^th ^case. Indications were predominantly for mammographic abnormalities (mass lesion & calcifications in 3 cases, non-specific area of enhancement in 1 case, and calcifications only in the remaining cases). An average of 9.1 (± 4.7) tissue cores per case was processed (1 to 25; median 8). DCIS was present in an average of 4.4 (± 2.4) tissue cores per case (1-11; median 4). DCIS ducts ranged in number from 4 to 117 (mean 26.4) in these cores. Cancerization of lobules by DCIS was identified in 23 (47%) of 49 cases. DCIS-associated calcification was present in 40 (81.6%) of 49 cases. A comedo-pattern DCIS (solid with central necrosis and nuclear grade 3/3) was present in 16 cases. The frequency and distribution of the various histologic patterns in the subset of cases associated with residual disease in the excision are outlined in Table 1.

**Table 1:** A comparison of biopsies and excisions regarding the frequency of selected pathologic features of DCIS**

**Morphologic features**	**Core biopsy** **(n = 40)**	**Excision** **(n = 40)**
Maximum nuclear grade 3	18	19
Maximum nuclear grade 2	22	21
Necrosis in DCIS	33	29
Calcification in DCIS	32	29
Comedo-pattern necrosis present (Solid pattern with central necrosis and grade 3/3 nuclear atypia)	16	14
Non-comedo pattern necrosis present (associated with grade 2/3 atypia and/or non-solid patterns)	17	16
Solid pattern only (irrespective of atypia or necrosis)	17	8
Solid and micropapillary only	0	1
Solid, micropapillary, and cribriform only	7	5
Micropapillary only	1	0
Micropapillary and clinging	1	0
Cribriform only	0	1
Micropapillary and cribriform only	2	1
Solid, micropapillary, clinging and cribriform only	0	3
Clinging only	0	0

DCIS ductal carcinoma in-situ; **excludes cases in which no residual DCIS was found in the excision.

### Excisions

Twenty (41%) and 29 (59%) of the excisions were products of mastectomies and various permutations of breast conserving surgeries (BCS), respectively. There was no residual carcinoma in 9 (18.4%) of 49 cases. Four (44.4%) of these 9 cases had changes diagnostic of atypical ductal hyperplasia (including one case that was re-classified from atypical lobular hyperplasia), whereas no atypical proliferations were present in the remaining five. The remaining 40 cases were comprised of specimens from 18 mastectomies and 22 BCS. The average specimen sizes (maximal dimensions) for the mastectomy and BCS-associated specimens were 19.6 cm (± 4.08) and 9.36 cm (± 2.5) respectively. A per-case average of 27 (± 11.9) tissue sections was processed from the 40 cases (10-60; median 24). For the 18 mastectomy specimens, an average of 24.6 (± 9.4) sections was processed per case (10-44; median 22.5). For the BCS-associated specimens, an average of 29.09 (± 13.4) sections was processed per case (12-60; median 25.5). Overall, an average of 7.52 (± 5) sections per case had DCIS (1-20; median 7), including an average of 6.4 (± 3.9) sections in the BCS-associated specimens and average of 8.9 (± 5.9) sections in the mastectomy specimens.

### Biopsies and excisions

There were 4 cases of invasive carcinoma and 3 examples of microinvasive carcinoma out of the 49 excision specimens that were evaluated, for an overall invasion frequency of 14%. The 4 cases included 1 mucinous and 3 ductal (not otherwise specified) carcinomas, and all were grade II/III by MSBR criteria. Axillary lymph nodes were also positive in 1 of these 4 cases (2 of 4 lymph nodes removed as part of sentinel node evaluation; 1 metastatic deposit > 0.2 cm and the other < 0.2 cm). Of the 28 cases in which a sentinel lymph node evaluation was performed, this was the only case with a positive node. As previously noted, in 5 (10.2%) of 49 cases, no residual carcinoma was found. In another 4 cases, the changes were diagnostic only of atypical ductal hyperplasia

Among the 40 cases with residual disease, the histologic patterns identified singly or in various combinations were micropapillary, clinging, cribriform, and solid - the latter including the comedo-pattern. There were 5 cases in which comedo-pattern DCIS was present in the excision but not in the core biopsy. The discrepancy was attributed to lower maximal nuclear grade in the biopsy proliferation in 4 cases and the absence of central necrosis in the 5^th^. When necrosis is considered in general (i.e. irrespective of whether it is specifically in association with the comedo-pattern), there were also 5 cases in which this finding was present in the excision but absent in the preceding biopsy. In 6 (15%) of the 40 pairs, calcification was present only in the excision specimens, For the other histologic patterns, in 8 (20%) of 40 cases, there were more histologic patterns identified in the core biopsy than in the corresponding excision. For the other 32 cases, there was general, albeit varying degrees of agreement between the core biopsies and the excisions. For the core histologic patterns, i.e. solid, micropapillary, cribriform, and clinging, 100%, 66%, 50%, 33% and 25% of the number of histologic patterns in the excisions were captured in 14 (35%), 2 (5%), 7 (17.5%), 6 (15%) and 3 (7.5%) of these 32 core biopsies respectively. Therefore, the core biopsy captured at least half of the core non-comedo histologic patterns in 77.5% of cases. These associations were not reflected upon statistical analysis: Pearson chi-square showed that among the histologic types of DCIS, only the presence of a cribriform pattern in the core biopsy significantly predicted the presence of a cribriform pattern in the excision (p = 0.028), with a Phi of 0.348, indicating a moderately strong association; this association did not hold with Fisher's exact test on the separate BCS and mastectomy groups

A general level of congruence between core biopsies and excisions was observed regarding maximal nuclear grade. Parenthetically, all 40 cases were either maximum nuclear grade 2 (n = 22) or 3 (n = 18). As such, there were 22 and 18 cases of intermediate and high-grade DCIS respectively. In 6 (15%) of the 40 cases, the maximum nuclear grade of the excision (grade 3) was higher than that seen in the core biopsy (grade 2). Pearson chi-square showed that maximum nuclear grade in the excision was significantly predicted by maximum nuclear grade in the core biopsy (p = 0.028), with a Phi of 0.347, indicating a moderately strong association. Again, this association did not hold with Fisher's exact test on the separate BCS and mastectomy groups. Other features that were evaluated, including calcification, necrosis, non-cribriform histologic pattern, and range of nuclear grades did not show a statistically significant correlation between the core biopsy and subsequent excision, either globally or within separate BCS and mastectomy groups, which may be related to our small sample size.

### Margin status in excisions and core biopsy features

The average clear margin width for all 40 cases was 0.63 cm (± 0.96; 0-4.5 cm; median 0.2 cm), including 6 cases with positive margins, 21 cases with clear margin width ≤ 0.2 cm, and 19 cases with clear margin width > 0.2 cm. For the 22 specimens associated with BCS, the average clear margin width was 0.35 cm (± 0.41; 0-1.5 cm; median 0.15 cm), including 5 cases with positive margins, 13 cases with clear margin width ≤ 0.2 cm, and 9 cases with clear margin width > 0.2 cm. For the 18 mastectomy-associated specimens, the average clear margin width was 0.97 cm (± 1.30; 0-4.5 cm; median 0.35 cm), including 1 case with positive margins, 8 cases with clear margin width ≤ 0.2 cm, and 10 cases with clear margin width > 0.2 cm. On univariate and multivariate analysis, the only core biopsy variable which significantly predicted a close margin on excision was the presence of necrosis (p = 0.027), with a Phi of 0.350, indicating a moderately strong correlation. This association did not hold when evaluated on separate BCS and mastectomy groups. No other significant association between core biopsy features, including maximum nuclear grade, comedo-pattern present, cancerization of lobules, number of tissue cores processed, DCIS ducts per tissue core, number of tissue cores with DCIS or other histologic patterns, and margin status was identified at the ≤ versus > 0.2 cm threshold (Table 2)

**Table 2:** Relationship between core biopsy features and margin status (univariate analysis)**

	Overall (n = 40)			Breast conserving surgery specimens (n = 22), margin status			Mastectomy specimens (n = 18), margin status		
Core biopsy features	≤ 0.2 cm (n = 21)	> 0.2 cm (n = 19)	**p**	≤ 0.2 cm (n = 13)	> 0.2 cm (n = 9)	**p**	≤ 0.2 cm (n = 8)	> 0.2 cm (n = 10)	**p**
Maximum nuclear grade 3/3 (n = 18)	9	9	**1**	3	3	**1**	6	6	**1**
Maximum nuclear grade 2/3 (n = 22)	12	10	**0.76**	10	6	**0.29**	2	4	**0.57**
Necrosis present (n = 33)	16	17	**1**	10	8	**0.74**	6	9	**0.47**
Comedo-pattern present (n = 16)	8	8	**1**	3	2	**NA**	5	6	**1**
Cancerization of lobules present (n = 21)	9	12	**0.54**	4	5	**1**	5	7	**0.68**
Number of tissue cores processed (mean)	8.6	9.4	**0.56**	9.4	8.4	**0.66**	7.25	10.3	**0.08**
DCIS ducts per tissue core (mean)	5.9	6.1	**0.85**	6	4.2	**0.17**	5.9	7.9	**0.35**
Number of tissue cores with DCIS	4.42	5.05	**0.45**	4.23	3.89	**0.68**	4.75	6.1	**0.31**
Total DCIS ducts (mean)	25.9	31.4	**0.49**	23.1	17.6	**0.35**	30.25	43.9	**0.4**
Solid Pattern present (n = 37)	20	17	**0.64**	13	7	**0.11**	7	10	**0.49**
Micropapillary pattern present (n = 10)	6	4	**0.66**	3	2	**NA**	3	2	**NA**
Cribriform pattern present (n = 18)	8	10	**0.53**	5	5	**1**	3	5	**0.62**
Clinging pattern present (n = 4)	3	1	**NA**	0	1	**NA**	3	0	**NA**

DCIS ductal carcinoma in-situ; **excludes cases in which no residual DCIS was found in the excision; NA not applicable

### Tumor size in excisions and core biopsy features

The average DCIS lesional size, as determined by multiplying 0.4 cm by number of blocks with DCIS [19], was 3.01 cm (± 1.99) (range 0.4-8 cm; median 2.8 cm). There was no significant difference regarding lesional size between the mastectomy specimens [mean 3.56 cm (± 2.34); median 3.6 cm] and the BCS-associated specimens [mean 2.56 cm (± 1.55); median 2.6 cm]. The average DCIS size was used to classify the 40 cases into two groups for analytical purposes. The first group was comprised of all cases with lesional size in the excision ≤ 2.7 cm (n = 22) whereas the lesional size was greater than 2.7 cm in the 18 cases that constituted the other group. At this size threshold, there was no significant association between lesional size in excisions and core biopsy features (number of DCIS ducts, DCIS ducts per tissue core, number of tissue cores with DCIS, and cancerization of lobules; Table 3). Furthermore, the clear margin width of the cases with lesional size ≤ 2.7 cm (mean 0.69 cm) was not significantly different from the cases with lesional size > 2.7 cm (mean 0.56 cm).

**Table 3:** Lack of significant correlation between selected core biopsy features and lesional size in excisions (at the ≤ 2.7 cm versus > 2.7 cm threshold)

**Core Biopsy features**	**Lesional size in excision ≤ 2.7 cm** **(n = 22)**	**Lesional size in excision > 2.7 cm** **(n = 18)**	**P**
Total DCIS ducts (means)	25.3	28.0	NS
Number of tissue cores with DCIS (means)	5.1	4.4	NS
DCIS ducts per tissue core (means)	5.01	6.3	NS
Cancerization of lobules present	12	8	NS

DCIS: ductal carcinoma in-situ

NS: Not significant

## Discussion

In the current environment, wherein screening-detected, calcification-associated, and impalpable DCIS are more frequently encountered, the core biopsy has emerged as an excellent modality for the preoperative establishment of the diagnosis and for the optimization of therapeutic options [20,21]. Nonetheless, the core biopsy is associated with a somewhat higher inaccuracy rate for the diagnosis of DCIS than for the diagnosis of invasive cancer [22,23]. In the study of Dillon et al [22], for example, the initial core biopsy was not fully diagnostic in 33% and 26% of their cases of intermediate and high-grade DCIS, respectively. Previous studies have evaluated the significance of DCIS size, extent, and histologic features in core biopsies as a predictor of margin status in the subsequent excisions [24-27]. However, these studies were centered on cases of DCIS admixed with invasive carcinoma. There is very limited published data on a potential correlation between the pathologic features of pure DCIS in core biopsies and margin status. The goals of the present study are two-fold: 1) To determine if there are any core biopsy morphologic features that may predict a close or positive margin in the subsequent excision in a group of intermediate and high-grade DCIS cases, and 2) To determine how well the core biopsy predicts the maximal pathology in the associated excisions.

Although there is no consensus of what constitutes a "close" margin [28], there is a near consensus that the status of margins after an excision for DCIS is a strong predictor of residual disease and/or local recurrence [29-34]. As such, there have been a variety of proposals that are aimed at establishing the adequacy of the excision intraoperatively, including intraoperative specimen ultrasonography, intraoperative specimen mammography, gross specimen examination, the routine sampling of the "cavity margins" around the initial excision, and frozen section evaluation of margins [35-39]. While mammographic features, such as linear branching calcification, accurately identify many examples of high-grade DCIS as such [40,41], this modality does not do so invariably [42], and may underestimate lesional size and extent, especially in non-comedo DCIS [43-45]. It may therefore be of clinical utility to have core biopsy features that may predict the necessity for a more generous excision during the initial surgery, which in addition to the aforementioned reasons, may also help stratify those patients that would qualify for specific therapies that require negative margins such as accelerated, hypofractionated whole breast radiotherapy [46] or partial breast intraoperative radiation [47]. Furthermore, in addition to histologic grade and margin status, it has been shown that several other pathologic features in the excision specimens may be associated with local recurrence [48-51]. Among these are volume of DCIS near margins and the overall DCIS volume in the specimen - whether deduced from the number of slides with DCIS, number of DCIS ducts or DCIS-cancerized lobules, or percentage of blocks with DCIS [48-51]. If any core biopsy features can predict a large volume in the excision, this may also be of clinical utility.

Our study shows that of all the core biopsy features that were evaluated, including maximum nuclear grade, necrosis, cancerization of lobules, number of tissue cores with DCIS, number of DCIS ducts per tissue core, total DCIS ducts, comedo-pattern or other histologic patterns, only necrosis was significantly associated with a positive or close margin on univariate and multivariate analysis. Similarly, at the 2.7 cm size threshold, there was no significant correlation between lesional size in excisions and selected core biopsy features (number of DCIS ducts, DCIS ducts per tissue core, number of tissue cores with DCIS, and cancerization of lobules). Most of the core biopsy features evaluated herein are therefore of limited value in predicting close or positive margins, or large lesional size in intermediate and high-grade DCIS. Our findings, however, need to be confirmed in substantially larger datasets from multiple institutions.

The second goal of this study was to determine how well the core biopsy predicted the maximal pathology in the patient, as evaluated in the subsequent excisions in this specific dataset of intermediate and high-grade DCIS. Regarding the risk of invasive disease in a core biopsy showing DCIS alone, previous studies of pan-grade DCIS have shown that invasive disease is present in 8 to 36% of cases [52-57]. In high-risk subsets, i.e. those patients with large masses, numerous mammographic calcifications, DCIS of high histologic grade, or presentation with a palpable mass, this risk is approximately 41.5 to 48% [58,59]. In our own dataset, we identified 4 examples of invasive carcinoma and 3 examples of microinvasive carcinoma out of the 49 excision specimens that we evaluated. Therefore, the risk of any kind of invasive disease in our patient population after a core biopsy diagnosis of intermediate or high-grade DCIS is somewhat low at 14% (7/49). There were too few cases of invasive disease to stratify for preoperative findings that may potentially predict invasion. The reverse corollary is that in 5 (10.2%) of 49 cases, no residual carcinoma was found. In another 4 cases, the changes were diagnostic only of atypical ductal hyperplasia. A recent report on 40,395 screening detected breast malignancies showed that in 174 cases no carcinoma was identified in the excision subsequent to the core biopsy [60]. In 165 of these cases, the authors concluded that the lesion was entirely removed in the core biopsy, whereas the remaining 9 were considered to false positive diagnoses in the biopsies [60]. Therefore, allowing for differences in sample size and composite of lesional types, our rate of finding no residual disease (5/49; 10.2%) is higher but largely comparable to the reported findings in that study (165/40,395; 4.3%).

The necessity for sentinel lymph node evaluation in patients with pure DCIS, and whether patient subsets can be preoperatively identified that would benefit the most from this evaluation, continues to be controversial [61,62]. Sentinel nodes are found to be positive in 4.8% to 13% of patients with a core biopsy diagnosis of DCIS [57,63-66]. Our findings provide insights into the frequency of lymph node involvement in this group of intermediate and high-grade DCIS. Amongst the subset of 28 patients that received any nodal evaluation, only 1 (3.6%) had sentinel lymph node involvement. Parenthetically, for this single patient, only touch preparations of the lymph node's cut section were performed intraoperatively, and did not reveal malignant cells even upon their detailed review in the aftermath. The malignant cells became morphologically evident only after subsequent additional node sectioning, when a "negative" intraoperative diagnosis had already been rendered. As such, she did not benefit from sentinel node evaluation. Our experience, therefore, is that the frequency of nodal involvement is low, which is concordant with most previously reported findings. However, our data is insufficient to make assertions about the validity of routine sentinel node evaluation in this group of patients.

We conclude that most of the core biopsy features evaluated herein are of limited value in predicting a close or positive margin in high and intermediate grade DCIS. A significant change [to invasive disease (14%) or to no residual disease (10%)] is seen in approximately 24% of excisions that follow a core biopsy diagnosis of intermediate or high-grade DCIS.

## Competing interests

The authors declare that they have no competing interests.

## Authors' contributions

OF wrote the initial version of the manuscript and participated in data collection and analysis. NFC performed data collection. MG performed data analysis and edited the manuscript. All authors read and approved the final manuscript.
